# A Comprehensive Approach to Parkinson’s Disease: Addressing Its Molecular, Clinical, and Therapeutic Aspects

**DOI:** 10.3390/ijms25137183

**Published:** 2024-06-29

**Authors:** Mauricio Muleiro Alvarez, Gabriela Cano-Herrera, María Fernanda Osorio Martínez, Joaquin Vega Gonzales-Portillo, Germán Rivera Monroy, Renata Murguiondo Pérez, Jorge Alejandro Torres-Ríos, Ximena A. van Tienhoven, Ernesto Marcelo Garibaldi Bernot, Felipe Esparza Salazar, Antonio Ibarra

**Affiliations:** 1 Centro de Investigación en Ciencias de la Salud (CICSA), Facultad de Ciencias de la Salud, Universidad Anáhuac Campus México Norte, Huixquilucan 52786, Mexico; 2 Facultad de Medicina, Universidad Peruana de Ciencias Aplicadas, Av. Alameda San Marcos 11, Chorrillos 15067, Peru; jovgagp95@gmail.com; 3 Secretaria de la Defensa Nacional, Escuela Militar de Graduados en Sanidad, Ciudad de México 11200, Mexico

**Keywords:** Parkinson’s disease, dopaminergic agonists, MAO inhibitors, antidiabetic agents, deep brain stimulation and levodopa/carbidopa intestinal gel infusion

## Abstract

Parkinson’s disease (PD) is a gradually worsening neurodegenerative disorder affecting the nervous system, marked by a slow progression and varied symptoms. It is the second most common neurodegenerative disease, affecting over six million people in the world. Its multifactorial etiology includes environmental, genomic, and epigenetic factors. Clinical symptoms consist of non-motor and motor symptoms, with motor symptoms being the classic presentation. Therapeutic approaches encompass pharmacological, non-pharmacological, and surgical interventions. Traditional pharmacological treatment consists of administering drugs (MAOIs, DA, and levodopa), while emerging evidence explores the potential of antidiabetic agents for neuroprotection and gene therapy for attenuating parkinsonian symptoms. Non-pharmacological treatments, such as exercise, a calcium-rich diet, and adequate vitamin D supplementation, aim to slow disease progression and prevent complications. For those patients who have medically induced side effects and/or refractory symptoms, surgery is a therapeutic option. Deep brain stimulation is the primary surgical option, associated with motor symptom improvement. Levodopa/carbidopa intestinal gel infusion through percutaneous endoscopic gastrojejunostomy and a portable infusion pump succeeded in reducing “off” time, where non-motor and motor symptoms occur, and increasing “on” time. This article aims to address the general aspects of PD and to provide a comparative comprehensive review of the conventional and the latest therapeutic advancements and emerging treatments for PD. Nevertheless, further studies are required to optimize treatment and provide suitable alternatives.

## 1. Introduction

**Figure 1:**
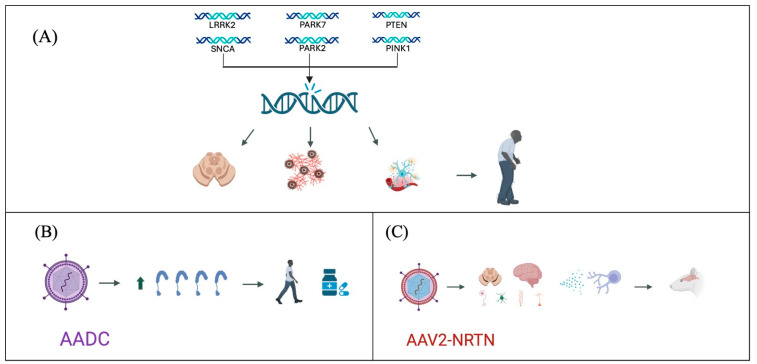
Advances in Parkinson’s disease genetic treatment strategies. (**A**) Genetic mutations identified as participants in the pathogenesis of Parkinson’s disease, leading to degeneration of dopaminergic neurons, accumulation of α-synuclein, and neuroinflammation. (**B**) Use of viral vectors for the expression of AADC, improving symptomatology and reducing pharmacological requirements. (**C**) Use of viral vectors for the expression of AAV2-NRTN, producing an anti-inflammatory and neurodegenerative environment, decreasing disease progression.

PD is the second most prevalent progressive neurodegenerative disorder after Alzheimer’s disease. Between the years 1990 and 2016, the prevalence of PD significantly increased from 2.5 million to 6.1 million cases globally, representing a 2.4-fold increase compared to the initial figure [1]. This disease entails a progressive impairment that notably impacts the lives of patients, their families, and caregivers, representing a high cost of treatment while disrupting their quality of life [2].

The primary risk factor for this disease is age, so as the population ages, the prevalence of this pathology is expected to increase as well. Some projections estimate that the population with PD will double by 2040, exceeding 12 million cases [3]. This pathology develops more frequently in men, representing 52.5% of cases compared to 47.5% in women. Similarly, individuals from countries with a medium to high socio-demographic index account for 85.2% of cases, contrasting with countries with a low socio-demographic index at 14.8% [1]. Likewise, some lifestyle-related risk factors have been identified, such as pesticide use, tobacco, coffee, physical activity, and a history of traumatic brain injury [4]. Additionally, viral microorganisms such as Herpes Simplex Virus-1, Ebolavirus, and Human Immunodeficiency Virus and bacteria such as Helicobacter pylori have been associated with PD [5]. However, the genetic component is the most notable risk factor, with more than 90 loci currently identified for this disease [6].

Traditionally, PD has been characterized by bradykinesia, postural instability, resting tremor, and rigidity [7]. The clinical presentation consists of non-motor and motor characteristics, with motor symptoms being part of the classic presentation. Non-motor symptoms include anxiety, sleep disorders, gastrointestinal symptoms, depression, olfactory loss, dysautonomia, and cognitive impairment. Both the clinical presentation and disease progression are heterogeneous and vary between each patient [8]. The onset of the disease tends to be slow, with an average period of 10 years between the initial symptoms and the medical approach or diagnosis [2,9]. The initial manifestations are characterized by being nonspecific, such as constipation, hyposmia, sleep disturbances, or depression, which contributes to the challenge of establishing an early diagnosis [10]. Numerous non-motor symptoms manifest well in advance of motor symptoms, leading to the emergence of the prodromal PD concept [11].

Moreover, its etiology is multifactorial, and the causes surrounding the disease are unique to each patient [2]. The characteristic histological features of PD consist of neuronal inclusions in the form of Lewy bodies and Lewy neurites, with cell loss in the substantia nigra and other brain regions [4]. Dopaminergic neurons in the substantia nigra pars compacta are killed, causing the characteristic motor symptoms of the disease [12].

Despite the availability of pharmacological, non-pharmacological, and surgical treatments, including brain, spinal, and vagus nerve stimulators, patients continue to experience progressive weakness, and no treatment has yet succeeded as a true disease modifier [13]. Unfortunately, regardless of research efforts related to PD, it remains in many aspects a topic with numerous knowledge gaps, lacking both prevention methods and a cure [14].

The aim of this study is to provide an extensive examination of the existing understanding of PD, diagnostic techniques, treatments, and investigational tools displaying promising outcomes for patients with the condition.

## 2. Pathophysiology

PD has a multifactorial etiology as it results from a combination of genomic, epigenetic, and environmental factors [15,16]. Multiple genes contribute to the disease’s onset and progression [15].

### 2.1. Genetics and Epigenetics

The most common mutations associated with PD occur in the *LRRK2* or *PARK8* gene, particularly among individuals aged 50 and above [17,18]. Several mutations have been described, the most common being *R1441H*, *R1441G*, *R1441C*, and *G2019S*, which cause a loss of dopaminergic neurons while altering processes such as cytoskeletal function, the lysosomal system, protein synthesis, and vesicle transport [17,18]. They also play a role in regulating inflammatory response and oxidative stress in the microglia [19,20].

*DJ-1* or *PARK7*, *PARK6*, and *PARK2* are the genes responsible for protecting dopaminergic neurons from hydrogen peroxide, rotenone, and mutant synucleins [21,22]. Therefore, a mutation in any of the above will increase oxidative stress, predisposing the patient to an increase in neurodegeneration. Another associated gene is *PINK1*, a mitochondrial kinase that accumulates on the membranes of damaged mitochondria, where it will recruit parkin to regulate mitophagy [23,24] and stop the collection of toxic products that cause neuronal loss [25]. A mutation in the aforementioned gene will cause symptoms and neuropathology characteristic of early-onset PD, accompanied by astrocytic gliosis and microgliosis [26].

One of the defining characteristics of PD is the reduction of dopaminergic neurons and the deterioration of the substantia nigra pars compacta. This deterioration has been closely linked to the excessive production of α-synuclein, a 140-amino acid protein present in the presynaptic terminals of neurons [7,27]. Predominantly localized in the substantia nigra, thalamus, neocortex, and cerebellum, α-synuclein is crucial for vesicle fusion and movement, axonal transport, neurotransmitter release, and synaptic connectivity. However, post-mortem examinations of individuals with PD have revealed that α-synuclein often misfolds, resulting in its accumulation within intracytoplasmic inclusions known as Lewy bodies [28,29]. These Lewy bodies are also found in other diseases related to PD. The intermediate molecules produced during this buildup contribute to harmful effects that ultimately impair synaptic function and cause neuronal degeneration [7,30].

The *SNCA* gene is responsible for encoding α-synuclein. *SNCA* mutations, mainly missense or multiplication variants, are associated with prompt and fast α-synuclein misfolding and aggregation [31]. Similarly, mutations in the *GBA* gene, responsible for encoding glucocerebrosidase, reduce its enzymatic function; this reduction leads to inadequate breakdown of α-synuclein and heightened exosomal release, ultimately contributing to the accumulation of Lewy bodies [32]. Controversially, the role of α-synuclein as the main triggering factor of PD has been questioned. Recent studies using novel monoclonal antibodies that inhibit α-synuclein aggregation have shown that, despite the elimination of these aggregates, parkinsonian symptoms persist and the disease continues to progress. This suggests that research should focus on identifying new pathophysiological targets for PD [33].

Recent research has shown that peripheral inflammation also plays a key role in the pathogenesis of PD. Along with a-synuclein, the neutrophil–lymphocyte ratio (NLR), lymphocyte–monocyte ratio (LMR), and neutrophil-to-high-density lipoprotein ratio (NHR) have been considered as useful peripheral inflammatory markers for neurodegenerative diseases. Neutrophils have the ability to penetrate epithelial cells on the blood vessel walls and mediate the inflammatory response by regulating chemokines [34]. Diverse studies correlate the role of NLR in PD, reporting that an increased NLR is associated with loss of neuronal connections and more pronounced motor impairment [35]. The study conducted by Madetko et al. evaluated the NLR and PLR in peripheral inflammation in PD using blood samples, and reported that patients with PD have higher NLR and PLR scores compared to the control group. Therefore, this can be used as a parameter for suspected PD [36].

As for lymphocytes, some studies carried out in mice have shown a correlation between higher lymphocyte count and less PD prevalence; therefore, the use of LMR is a useful marker for this condition. Moreover, high levels of high-density lipoprotein cholesterol are associated with the integrity of the blood–brain barrier, and, in patients with PD, the concentrations are significantly lower. The NHR is negatively related to disease duration [37]. A study carried out by Li et al. showed that elevated plasma NLR and NHR, as well as low concentrations of LMR, were closely related to the severity of the disease, hence their novel clinical importance in the diagnosis and prognosis of PD; however, more research is needed to fully validate these results [34].

The platelet–lymphocyte ratio (PLR) is calculated by dividing the number of platelets by the number of lymphocytes obtained from a blood sample. This parameter is used to differentiate PD from essential tremor, since platelets participate in the inflammatory response. The study conducted by Madetko et al. evaluated the NLR and PLR in peripheral inflammation in PD using blood samples, and reported that patients with PD have higher NLR and PLR scores compared to the control group. Therefore, this can be used as a parameter for suspected PD [36].

### 2.2. Mechanisms of Neural Damage

#### 2.2.1. Neuroinflammation

Further neuroinflammatory mechanisms have been suggested as potential factors influencing the development of PD. Numerous apoptotic nigrostriatal dopaminergic neurons have shown increased levels of molecules such as IL-6 and TNF-α, as well as apoptosis-related factors including caspases 1 and 2, Fas, and p55 [15,27]. Oxidative stress is another mechanism that directly harms the central nervous system (CNS), resulting in cell death, protein degradation, DNA damage, and enzyme malfunction in various neurons, including dopaminergic ones [38]. Reactive oxygen species (ROS), a critical component of oxidative stress, arise from ineffective oxidative phosphorylation in the electron transport chain, leading to mitochondrial dysfunction. The decline of dopaminergic neurons due to ROS is linked to reduced glutathione levels and elevated levels of metals such as iron and calcium in the substantia nigra pars compacta [39].

#### 2.2.2. Microglial Activation

Microglial activation is another pathway for chronic cytokine and ROS production. The main source of ROS in this context is NADPH oxidase, which accelerates dopaminergic neuronal death [40].

#### 2.2.3. Neurovascular Unit

As observed in various other neurodegenerative diseases, the neurovascular unit plays a significant role in the progression of PD [41]. Studies conducted on mice with induced PD suggest notable changes in the blood–brain barrier, which may allow the passage of harmful macromolecules and neurotoxins. Consequently, the breakdown of the blood–brain barrier may contribute to dopaminergic neuron loss [42]. Additionally, since L-DOPA is essential for dopamine synthesis, it has been suggested that blood–brain barrier dysfunction affects the L1 transporter, a protein found in the cell membranes of neurons, resulting in a deficiency of the neurotransmitter [43].

#### 2.2.4. Gut–Brain Axis

The growing interest in microbiota research has shed light on the close relationship between intestinal eubiosis and neuroinflammatory processes, commonly referred to as the gut–brain axis. Disruption in the regulation of the enteric system may play a role in the development of PD, potentially due to heightened permeability of the intestinal epithelial barrier. This hypothesis is strongly supported by observed alterations in the microbiomes of patients with PD, which include a decrease in the abundance of genera such as Prevotella, Roseburia, and Blautia—all belonging to the family Lachnospiraceae. Conversely, there is a notable increase in the family Lactobacillaceae, which includes bacteria recognized as proinflammatory. These changes, facilitated by retrograde conduction, are potential causes of neurotoxicity and chronic neuroinflammation [44].

#### 2.2.5. Ferroptosis

Additional proinflammatory metabolic processes have been linked to the development of PD. A notable example is abnormal iron deposition. Ferroptosis, a form of cell death, depends on disrupted iron metabolism, leading to oxidative stress and ultimately cell death [45]. Accumulated iron in the substantia nigra binds to α-synuclein with affinity, promoting its cytotoxic aggregation and increasing the number of reactive oxygen species (ROS) [46]. Ultimately, it is established that PD does not have a single cause or trigger. It is recognized as a neurodegenerative disease typically occurring later in life, with a significant genetic component that manifests in susceptible individuals exposed to epigenetic changes.

## 3. Diagnostic Methods

Due to the non-motor symptoms that often precede motor manifestations, nearly 10% of patients are misdiagnosed with other pathologies [47]. Consequently, the International Parkinson and Movement Disorders Society established specific criteria to improve diagnostic accuracy. According to their recommendations, the diagnosis of PD necessitates the presence of bradykinesia and at least one of the following symptoms: resting tremor (4–6 Hz) or limb rigidity. Moreover, it is crucial to consider exclusion criteria to eliminate PD as a diagnosis and meticulously evaluate alarming data that could indicate potential signs of other pathologies [48].

In addition to detailed questioning and precise physical assessment, several resources aid in the diagnosis of PD. Imaging techniques, such as dopamine transporter single-photon emission computed tomography (DAT-SPECT) and structural magnetic resonance imaging (MRI), are commonly utilized due to their specificity. MRI, in particular, provides distinctive characteristics useful for identifying atypical parkinsonism, including neuromelanin imaging (NMI), which detects changes in the substantia nigra pars compacta and the locus coeruleus [38].

Genetic studies are typically reserved for cases suspected of having a hereditary basis for PD. However, it is increasingly recognized that such studies should be considered routine, as the identification of specific genes associated with severe PD symptoms has been linked to a poorer prognosis [38].

## 4. Pharmacological Treatments

### 4.1. Conventional Treatment

#### 4.1.1. Levodopa and Its Derivatives

Several treatment options are available for PD, with a history of evolving therapies aimed at alleviating patients’ symptoms over time. Dr. Gerald Stern, a pioneering figure in neurology in the UK, achieved a significant medical breakthrough in 1930 by introducing the use of L-dopa for PD patients. Additionally, he contributed to the adoption of direct-acting dopamine agonists (DAs) and the application of monoamine oxidase inhibitors (MAOIs) [49]. Guidelines recommend initiating treatment for motor symptoms when they significantly impact patients’ quality of life [50].

Dopamine, levodopa formulations, monoamine oxidase B inhibitors (MAOIs), and dopamine agonists (DAs) serve as valuable initial therapies for managing motor symptoms, especially in younger patients with prominent tremor. Among patients treated with oral dopamine agonists, approximately 40% may develop impulse control disorders, while about 20% may experience withdrawal symptoms upon discontinuation of DA treatment [15].

Levodopa can effectively cross the blood–brain barrier to convert into dopamine, which is then stored in the presynaptic terminals of striatal neurons [15]. This medication is highly efficient in the initial years of use, as there are adequate dopamine neurons capable of storing dopamine and regulating its release in the striatum [51]. However, as the disease advances, long-term use of levodopa can lead to dyskinesias that significantly impact patients’ quality of life [52]. Levodopa therapy effectively alleviates motor symptoms, notably tremor, bradykinesia, and rigidity. Its beneficial effects on tremor encompass both resting and postural types; however, it poses an increased risk of inducing dyskinesias and motor fluctuations [15,53,54,55]. However, its efficacy relies on the action of carbidopa, a decarboxylase inhibitor that prevents the peripheral metabolism of levodopa into dopamine. This mechanism reduces peripheral dopamine levels and mitigates associated adverse effects such as nausea, vomiting, and cardiac arrhythmias [15].

#### 4.1.2. Monoamine Oxidase B Inhibitors (MAOI-Bs)

MAOI-Bs are considered first-line medications for younger patients with mild motor symptoms at the onset of PD. They work by inhibiting the degradation of levodopa, thereby aiming to prolong and enhance its impact on dopaminergic neurotransmission. While MAOI-Bs improve motor symptoms, their efficacy is somewhat lower compared to levodopa; however, they carry a reduced risk of inducing dyskinesia. Consequently, PD patients often undergo treatment involving multiple drug classes to maximize benefits and minimize adverse effects. As the disease progresses, the brain’s capacity to store excess dopamine diminishes, resulting in a decreased response to medications and necessitating higher doses over time [10,53,56]. Pirker and colleagues have observed that combining levodopa with MAOI-Bs improves management of motor symptoms [53]. Non-ergot dopamine agonists, such as pramipexole, rotigotine, and ropinirole, are less potent than carbidopa in managing motor symptoms but offer the advantage of not inducing dyskinesias and motor fluctuations. Despite being more effective than MAO-B inhibitors in managing motor symptoms, non-ergot dopamine agonists can cause side effects such as hallucinations, impulse control disorders, and drowsiness [57,58].

#### 4.1.3. Catechol-O-Methyltransferase Inhibitors (COMT-Is)

Catechol-O-methyltransferase (COMT) is present in both the central nervous system (CNS) and peripheral tissues. It plays a role in accelerating the conversion of levodopa to 3-O-methyl-DOPA (3-OMD), thereby decreasing levodopa levels. 3-OMD competes with levodopa for transport across the blood–brain barrier and for conversion into dopamine in the CNS, potentially exacerbating motor symptoms [59].

COMT inhibitors (COMT-Is) work by increasing levodopa levels in the CNS directly and by reducing the presence of its competitor, 3-OMD. By enhancing the dopaminergic effects of levodopa, COMT-Is can induce adverse effects in the CNS such as drowsiness, confusion, dyskinesia, hallucinations, and depression, as well as gastrointestinal issues like diarrhea and colitis [60].

Currently, three COMT inhibitors are FDA-approved as adjunctive treatments for PD. Tolcapone was the first COMT inhibitor to receive FDA approval, in 1998. It effectively inhibits COMT function both peripherally and centrally. However, due to its association with liver failure, tolcapone is rarely used today and is recommended only as adjunctive therapy for patients with predominant motor symptoms who have not responded adequately to conventional treatments.

Entacapone was the second COMT inhibitor approved by the FDA, in 2003. It works by slowing down the peripheral metabolism of levodopa, thereby increasing its bioavailability and prolonging its action [59].

Opicapone, the third COMT inhibitor to be approved, functions by inhibiting the peripheral metabolism of levodopa. This action extends levodopa’s half-life and enhances its ability to deliver dopamine to the brain, thereby improving motor symptoms. However, there is currently no evidence in the literature supporting its ability to slow down the progression of PD. Abrupt discontinuation of opicapone may lead to withdrawal symptoms such as confusion and hyperpyrexia [56].

#### 4.1.4. Anticholinergic Agents

Anticholinergic agents, including trihexyphenidyl, benztropine, and amantadine, were among the earliest pharmacological treatments developed for PD and are typically considered for initial therapy in patients younger than 65 years who primarily exhibit tremor. Their primary effect is reducing tremors. However, due to their limited effectiveness and potential adverse effects, their use is restricted both as standalone treatments for symptomatic patients with dyskinesia and as adjuncts to levodopa in stable PD.

Amantadine is a dopamine agonist that works by blocking N-methyl-D-aspartate (NMDA) receptors, thereby inhibiting dopamine reuptake and increasing dopamine release from presynaptic neurons [15,53]. It is indicated for reducing off time without exacerbating dyskinesias, due to its effects on various neurotransmitter systems including cholinergic, serotonergic, and noradrenergic systems [61].

These medications may cause side effects such as blurred vision, urinary retention, and altered sweating. However, the selection of medication is guided by considerations of patient safety, preferred route of administration, and cost-effectiveness [53,62].

### 4.2. Nonconventional Treatment

#### 4.2.1. Antidiabetic Agents

Considerable evidence suggests a connection between metabolic disorders and neurodegenerative conditions such as PD. Alterations in insulin signaling, a hallmark in adults with type 2 diabetes mellitus (T2DM), have also been observed in individuals with PD. Epidemiological studies indicate an increased risk of PD among those with T2DM. A meta-analysis by Yue et al., involving 1,761,632 subjects, demonstrated that T2DM elevates the risk of developing PD by nearly 40% [63,64].

Beyond serving as a risk factor, T2DM has been shown to worsen disease progression and increase symptom severity in PD [65]. There are shared pathological mechanisms between these diseases, including neuroinflammation, oxidative stress, and insulin resistance. Antidiabetic medications may potentially offer anti-inflammatory and neuroprotective effects in patients with PD who also have T2DM [66,67,68].

#### 4.2.2. Intranasal Insulin

Insulin receptors are distributed throughout the basal ganglia, underscoring the role of insulin in regulating neuronal growth, maintaining synapses, and neurotransmission [69]. Research indicates that intranasal insulin (INI) administration can prevent the loss of dopaminergic neurons, thereby enhancing motor function in PD [69]. Moreover, it has demonstrated efficacy in reducing dopamine-dependent cognitive impairment by restoring insulin signaling pathways without affecting peripheral glucose levels [70].

In a placebo-controlled clinical study conducted by Novak et al., involving 15 patients with PD, administration of 40 IU of INI was shown to improve motor functions and preserve cognitive functions [71].

#### 4.2.3. DPP-4 Inhibitors and GLP-1 Agonists

Glucagon-like peptide 1 (GLP-1) is a hormone with receptors located in the central nervous system (CNS) that has demonstrated the ability to inhibit apoptosis and improve cell survival. The enzyme dipeptidyl peptidase 4 (DPP-4) is responsible for metabolizing and clearing GLP-1 from the bloodstream [72].

Medications that target these pathways include GLP-1 receptor agonists, which mimic the effects of GLP-1, and DPP-4 inhibitors, which prevent its degradation. These medications exert an incretin effect, which includes reducing glucagon secretion, enhancing glucose-dependent insulin release, delaying gastric emptying, and promoting satiety [73].

A study carried out by Foltyine et al. has shown the potential utility of DPP-4 inhibitors and GLP-1 receptor agonists in the context of neurodegenerative diseases. In PD, they exert their neuroprotective effect thanks to their anti-inflammatory and antiapoptotic properties, thus improving motor and cognitive performance [72]. Similarly, these compounds have been shown to impact mitochondrial biogenesis, promote neurogenesis, and restore insulin signaling within the CNS [74].

In a cohort study conducted by Brauer et al., it was observed that the incidence of PD was 36–60% lower among individuals treated with DPP-4 inhibitors and GLP-1 agonists compared to those using alternative antidiabetic medications [75]. A 2020 meta-analysis by Wang et al. indicated that the administration of exenatide, a GLP-1 receptor agonist, was associated with enhanced performance in cognitive assessments, a reduction in non-motor symptoms, improvement in motor symptoms, and an overall enhancement in quality of life [76].

#### 4.2.4. Biguanide

Metformin, an oral hypoglycemic agent from the biguanide family, is widely used globally for treating T2DM. This medication acts on multiple sites to lower glucose levels. It inhibits hepatic glucose production by reducing gluconeogenesis and glycogenolysis, decreases glucose absorption in the intestine, and enhances glucose uptake in muscles, while also increasing the presence of GLP-1 [77].

Metformin’s use in neurodegenerative diseases is debated due to conflicting literature. Some studies suggest it may increase the risk of cognitive impairment and neurodegenerative diseases, potentially due to its association with cobalamin (vitamin B12) deficiency [78,79]. Vitamin B12 is crucial as a cofactor for enzymes involved in genetic material synthesis, fatty acid metabolism, and amino acid synthesis, as well as for its neuroprotective role and contribution to cell maturation in bone marrow and myelin synthesis [80].

However, a study by Sluggett et al. concluded that long-term use of metformin is not a risk factor for PD and may even reduce its incidence. This finding aligns with other research indicating the anti-inflammatory and neuroprotective effects of metformin [81,82,83,84].

## 5. Non-Pharmacological Treatments

### 5.1. Personalized Medicine

#### 5.1.1. Pluripotent Stem Cells

Recent advancements in genetics and regenerative medicine have spurred the quest for innovative therapies aimed at halting or slowing the advancement of specific neurodegenerative disorders through the utilization of pluripotent stem cells, which represent a promising alternative for cellular therapy in localized neurodegeneration, such as PD [85]. Although PD involves dysfunction in multiple systems and neurotransmitters leading to similar symptoms, the highly variable responses observed in patients receiving L-dopa treatment suggest the presence of diverse biochemical degeneration mechanisms. This underscores the need for alternative therapeutic options. Personalized medicine holds promise for delivering more effective treatments tailored to individual patients [86,87].

Some genetic factors may be implicated in the heterogeneity of the response to drugs used in PD [88]. Multiple studies have explored the potential role of single-nucleotide polymorphisms in individuals with PD and in their response to pharmacological treatments. One such gene of interest is the dopamine active transporter 1 (*DAT1*) gene, which plays an important role in dopamine reuptake in synapses; it also carries a higher risk of causing patients to develop dyskinesias when receiving L-dopa treatment and to experience hallucinations when exposed to dopaminergic drugs [89].

Regarding novel cell therapy advancements, pluripotent stem cells have emerged as promising targets in models of human diseases and regenerative medicine. The primary challenge of this therapeutic approach lies in precisely directing the differentiation of pluripotent stem cells into specific cell lineages. In the context of PD, this involves generating dopaminergic cells specific to the mesencephalon through directed differentiation technologies, crucial for producing these essential cell types [90].

For many years, researchers have investigated the transplantation of fetal ventral mesencephalic cells as a treatment for PD patients. This cell population includes dopaminergic, oculomotor, and retinal neurons [91]. A study carried out by Li et al. examined post-mortem brains from patients who received fetal cell transplants, revealing significant growth and innervation of the striatum by the transplanted cells. They also demonstrated that these neurons can survive for over 20 years in the human brain [91]. However, some patients who underwent transplantation developed graft-induced dyskinesia [90,92]. Since then, efforts have persisted in developing standardized protocols for cellular transplants, which are currently under study [93].

#### 5.1.2. Gene Therapy

Despite PD being considered an idiopathic disease, several associated genes include *ATP13A2*, *dardin*, *DJ-1*, *alpha-synuclein*, *leucine-rich repeat kinase 2* (*LRRK2*), and *PINK1* [16,94]. Additional mutations are found in the *GBA1* gene, which is responsible for encoding the glucocerebrosidase enzyme. These mutations lead to neurotoxicity and neuroinflammation, influencing the age of onset, severity of symptoms, and progression of cognitive impairment [95].

Gene therapy has emerged as a therapeutic option for correcting defective genes and introducing therapeutic genes in several diseases. In PD, various gene therapy methods are being explored in both animal models and humans, showing promising results. The objectives of this treatment approach are categorized into disease modifiers and non-disease modifiers [96].

Regarding non-disease modifiers, studies in rodents have utilized adenovirus vectors to overexpress Aromatic L-Amino Acid Decarboxylase (AADC), which is decreased in patients with PD. This approach has demonstrated improvements in parkinsonian symptoms and a reduction in the need for medication in cases of advanced disease [97].

Regarding disease-modifying treatments, studies have investigated infusions of adeno-associated virus-neurturin (AAV2-NRTN) in the striatum, caudate nucleus, and putamen. These studies have shown preservation of nigral neurons and an increase in Tyrosine hydroxylase-positive (TH+) cells, suggesting a potential reduction in disease progression [98]. Additionally, research has explored the use of neurotrophic factors such as glial cell line-derived neurotrophic factor (GDNF), which has been associated with an increase in TH+ cell counts, thereby mitigating the loss of dopaminergic neurons and potentially slowing disease progression [99] (Figure 1).

Despite promising results in animal studies, gene therapy for PD faces challenges in clinical translation. Human studies have not yielded expected outcomes, primarily due to issues related to gene distribution [99,100,101,102,103]. Therefore, while gene therapy represents significant progress in PD treatment, it currently remains an impractical option until further clinical studies confirm its efficacy and safety.

## 6. Surgical Treatment

### 6.1. Ablative or Lesioning Procedure

Pharmacological treatment effectively controls PD symptoms and improves quality of life. However, long-term use at high doses can lead to refractory symptoms and drug-induced side effects in some patients [104]. In cases where pharmacological treatment fails to achieve its objectives, surgical options become a viable alternative [105]. Surgical approaches are tailored to individual needs and may include unilateral procedures like thalamotomy, pallidotomy, or subthalamotomy, along with deep brain stimulator (DBS) placement, aiming to enhance relief of motor symptoms [106,107].

Confirming a PD diagnosis is crucial before considering surgical intervention, as other conditions can mimic its clinical features. Relative contraindications for fetal cell transplantation in PD include severe gait disturbances and postural instability. Neurosurgical treatment is primarily indicated for patients with moderate to severe disease who respond to levodopa pharmacological treatment but experience motor symptom fluctuations affecting daily life independence [106].

The response to levodopa treatment serves as a predictive indicator for surgical outcomes, typically measured by a minimum of 30% improvement on the Unified Parkinson’s Disease Rating Scale (UPDRS III) or Movement Disorder Society Unified Parkinson’s Disease Rating Scale (MDS-UPDRS III). Patients who do not respond adequately to levodopa generally do not benefit from surgical interventions, except in cases involving tremor improvement. For patients who experience some benefit from low-dose levodopa but cannot tolerate higher doses due to drug-induced secondary symptoms like nausea, dystonia, or dyskinesias, surgical options provide an alternative with potential positive outcomes. Fetal cell transplantation is considered for functional patients in early PD stages to help sustain their quality of life and daily activities [105,106].

Lesioning surgeries involve the targeted destruction of specific brain areas to disrupt affected neuronal networks [104]. Examples include unilateral thalamotomy, pallidotomy, and subthalamotomy. Unilateral thalamotomy may alleviate medication-resistant tremor in certain PD patients but does not improve bradykinesia, dyskinesia, or motor fluctuations. Unilateral pallidotomy is effective and safe for severe dyskinesia and motor fluctuations, with the benefit of sustained suppression of contralateral dyskinesia over many years. Unilateral subthalamotomy, however, carries a higher risk of adverse neurological effects, such as persistent dyskinesia, and is thus considered an experimental treatment for advanced PD [108].

Unilateral thalamotomy using MRI-guided focal ultrasound is an innovative method for treating tremor in PD. During this procedure, the patient is positioned in a stereotactic frame with an MRI-compatible ultrasound transducer [109]. Real-time MRI imaging guides the orientation of the transducer, and incremental doses of ultrasound energy are administered until the target area achieves therapeutic temperatures for lesioning. The effectiveness of the procedure is evaluated intraoperatively through clinical assessments of tremor while the patient remains awake [106].

Radiosurgery is another non-invasive therapeutic approach that employs high doses of ionizing radiation to selectively target and disrupt specific areas of the nervous system. This technique aims to alleviate symptoms by focusing on regions such as the ventral intermediate nucleus (VIN), subthalamic nucleus, and internal globus pallidus (GPi). However, radiosurgical pallidotomy has shown less favorable outcomes compared to other targets [106,110].

### 6.2. Deep Brain Stimulation (DBS)

DBS is a widely utilized surgical intervention for managing the motor symptoms of PD, including bradykinesia, tremor, and rigidity [111,112]. This procedure involves implanting small electrodes with a quadripolar configuration, typically made from platinum–iridium with nickel connectors and wrapped in polyurethane, to deliver controlled electrical impulses to specific brain regions. DBS effectively reduces dyskinesias associated with medication use by decreasing the reliance on pharmacological treatments.

The primary targets for DBS are the GPi and the subthalamic nucleus (STN). Randomized controlled trials have demonstrated motor improvement ranging from 25% to 60% with stimulation of either GPi or STN. GPi stimulation is particularly effective in reducing motor complications, whereas STN stimulation not only reduces the need for dopamine replacement medications but also enhances motor symptoms [107].

One of the challenges with DBS is the lifespan of the battery, which represents a significant limitation. Recent advancements include the development of rechargeable devices that can adapt to the patient’s needs by utilizing local field potentials (LFPs) from cortical or subcortical locations, thereby improving the durability of the device [110,111].

Stimulation-induced effects such as changes in balance, changes in gait, dysarthria, and dyskinesias can occur, along with non-motor changes like alterations in sleep patterns and cognitive impairment. These effects necessitate careful adjustment of the stimulation parameters to optimize therapeutic benefits while minimizing side effects [107,111].

### 6.3. Focused Ultrasound Stimulation (FUS)

FUS is a minimally invasive technique that uses targeted ultrasound waves to induce therapeutic chemical, thermal, or mechanical changes in deep tissues. Operating at higher frequencies and intensities, FUS concentrates energy on specific areas, enabling precise and localized treatment with minimal damage to surrounding tissues. This advanced technology provides a safer and more precise alternative to invasive surgical procedures [113,114].

In the context of PD, FUS, particularly through high-intensity focused ultrasound (HIFU), has shown significant promise. Unlike traditional diagnostic ultrasound, HIFU employs high-energy pulses to target tissues precisely, minimizing collateral damage [115]. This non-invasive approach is effective for neuromodulation, thermal ablation, and enhanced drug delivery. FUS offers a non-ionizing and reversible method to modulate deep brain structures, potentially alleviating motor symptoms and improving the quality of life for PD patients. Ongoing research and clinical trials aim to confirm the safety and efficacy of FUS, establishing it as a viable, less invasive alternative to conventional treatments such as deep brain stimulation [114,115].

### 6.4. Gamma Knife Thalamotomy (GKT)

GKT is a minimally invasive neurosurgical procedure used to treat refractory tremors, including those associated with PD. It delivers precise gamma radiation to the ventral intermediate nucleus of the thalamus, disrupting aberrant neural circuits responsible for tremor generation. GKT offers advantages such as minimal invasiveness, avoidance of risks inherent in open surgery, and suitability for patients ineligible for DBS. Clinical evidence supports GKT’s safety, with no significant long-term cognitive, gait, or speech impairments reported. Risks include transient side effects like headache and nausea, as well as the potential for radiation-induced neurologic changes, underscoring the need for careful patient selection and continuous monitoring [116,117,118].

## 7. Continuous Infusion

### 7.1. Levodopa/Carbidopa Intestinal Gel Infusion (LCIG)

For patients experiencing fluctuations in motor symptoms due to conventional levodopa/carbidopa (LC), often manifesting as the “on–off phenomenon”, an alternative therapeutic approach known as levodopa/carbidopa intestinal gel infusion (LCIG) is available [119].

LCIG involves a surgical procedure where a system continuously delivers carbidopa and levodopa infusion via a percutaneous endoscopic gastrojejunostomy using a portable infusion pump. This method offers several advantages over oral administration. First, it allows for continuous medication delivery directly to the jejunum, bypassing gastric emptying issues associated with oral intake. This continuous infusion ensures uninterrupted distribution of levodopa and carbidopa, leading to more stable medication concentrations in the bloodstream [119]. The stability in medication levels achieved through LCIG therapy results in increased “on” time and decreased “off” time for patients. This improvement in symptom control enhances their quality of life by minimizing fluctuations and reducing the severity of both motor and non-motor symptoms associated with PD [120].

In a study conducted by Wiredefeldt et al., continuous intestinal infusion therapy significantly reduced “off” time in patients with PD and, to a lesser extent, reduced dyskinesias [121]. This finding was corroborated by van Wamelen et al. in their review, where they observed a reduction in “off” time by 1.9 h per day and an increase in “on” time by 2.28 h per day without causing dyskinesias compared to oral levodopa therapy [122]. However, a study by Miyaue et al. reported that while infusion therapy extended “on” time, it did not reduce dyskinesias [123].

**Figure 2:**
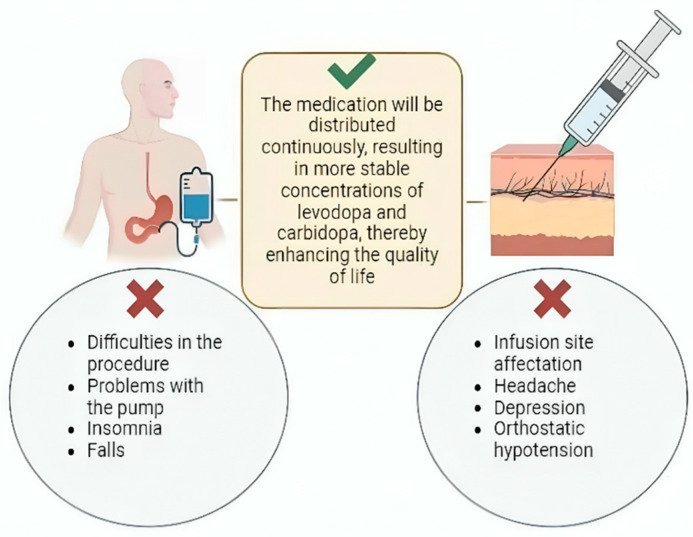
Continuous infusion treatment. Continuous LC infusion can be achieved through two mechanisms: LCIG and subcutaneous continuous infusion. Both promote more stable concentrations of these drugs. However, among their main complications are problems with both procedures, pump malfunctions, insomnia, falls, headache, depression, and orthostatic hypotension.

Despite its benefits, the use of infusion pumps for PD treatment is associated with several adverse effects. A comprehensive study by Lang et al., which analyzed multiple trials involving 412 patients over varying durations, reported that 76% of patients experienced adverse effects related to the procedure or the pump. Additionally, 17% experienced serious adverse effects unrelated to the pump or procedure. Among these, insomnia (23%) and falls (23%) were the most frequently reported non-pump-related complications. Notably, 17% of patients discontinued pump use due to adverse effects [124] (Figure 2).

New studies are needed to clarify the future directions of the LCIG, which could include the development of a smaller pump, the introduction of new medications, and, above all, the reduction of associated adverse effects so that the patients can have a better therapeutic option compared to conventional treatments.

### 7.2. Subcutaneous Infusion

Another viable method for administering a continuous dose of levodopa/carbidopa (LC) is through a subcutaneous approach, which has shown promising results in clinical studies. Rosebraugh et al. explored the combination of foslevodopa/foscarbidopa (ABBV-951), a prodrug with a solubility 100 times greater than LC. This enhanced solubility enables higher concentrations to be administered subcutaneously, making it a minimally invasive option [125].

Similarly, Giladi et al. investigated the safety and pharmacokinetics of ND0612, a continuous subcutaneous administration system under development. This system allows for the delivery of smaller volumes, resulting in stable plasma levels of LC without invasive procedures in patients experiencing motor fluctuations due to PD. These advancements highlight the potential of subcutaneous LC therapies to provide consistent medication delivery and improve treatment outcomes for PD patients [126].

Furthermore, LeWitt et al. conducted a study comparing the concentrations of ND0612 and placebo in patients with Parkinson’s disease (PD) as well as in healthy controls. They demonstrated that continuous subcutaneous infusion of levodopa/carbidopa (LC) at concentrations of 60 mg/mL and 7.5 mg/mL, supplemented with oral treatment, is suitable for continuous 24-h administration in patients with PD [127].

These systems offer several advantages, primarily achieving more stable drug levels through a minimally invasive approach. The studies highlighted a decrease in “off” time and an increase in “on” time with greater solubility and higher drug concentrations, leading to reduced fluctuations in symptom control. Importantly, they also demonstrated effectiveness and safety with minimal adverse effects. These findings suggest that despite being in development, continuous subcutaneous LC infusion systems are a viable alternative for PD patients experiencing motor symptoms [125,126,127].

### 7.3. Apomorphine Subcutaneous Infusion

Apomorphine is a dopamine receptor agonist known for its potent effect and high affinity for D1 and D2 receptors, as well as 5HT2 and α-adrenergic receptors [128,129]. Originally discovered by Matthiessen and Wright in 1869, it has since been extensively studied and utilized in treating various central nervous system disorders.

At low doses, apomorphine suppresses the firing rate of nigrostriatal neurons and reduces locomotor activity by presynaptically inhibiting endogenous dopamine release [128]. Its therapeutic uses have spanned a wide range of conditions, including as an emetic (to induce vomiting), sedative, and hypnotic, and for managing symptoms in disorders such as alcohol addiction-related delirium, schizophrenia, mania, depression, insomnia, chorea, Jacksonian epilepsy, and erectile dysfunction [130].

Apomorphine was first administered subcutaneously to treat PD in low doses, demonstrating antiparkinsonism effects with improvement in rigidity, muscle weakness, and tremor lasting up to three hours. However, adverse effects such as emesis, nausea, orthostatic hypotension, azotemia, skin reactions at the injection, and, in rare cases, eosinophilic panniculitis or Coombs positive hemolytic anemia were also reported [128,129,130]. The low oral bioavailability and short half-life of apomorphine, combined with its adverse effects when given in higher doses, led to the development of a long-lasting formulation in the form of subcutaneous acute injections or continuous subcutaneous infusion via a micropump [131].

The use of apomorphine has demonstrated a reduction by up to 80% in “off” time in patients through a portable pump system, effectively increasing its half-life [129,130]. Several studies have confirmed apomorphine’s efficacy in reducing “off” time and managing levodopa-related motor fluctuations in PD patients, whether administered through subcutaneous intermittent injections or continuous infusion, showing similar antiparkinsonism efficacy to levodopa [129,130].

The TOLEDO study, a multicentric phase 3 trial, compared apomorphine subcutaneous infusion to placebo in PD patients with persistent levodopa-related motor fluctuations despite optimized medication. Patients receiving apomorphine subcutaneous infusion showed a significant reduction in “off” time by over two hours, along with an increased time spent without troublesome dyskinesia, a reported sense of improvement, and a reduction in the need for concomitant oral therapy. The TOLEDO study results underscore the role of apomorphine as a viable therapeutic option for patients with advanced PD and severe levodopa-associated motor complications [128,129].

## 8. Life Changes

### 8.1. Exercise

Non-pharmacological treatments, such as physical activity, are recommended to enhance functional capacity, slow disease progression, and improve motor skills in patients with PD. Rehabilitation and physiotherapy are integral for addressing both non-motor and motor symptoms, while also potentially slowing disease progression. Exercise programs typically include resistance and aerobic exercises incorporating hand movements [132]. A retrospective observational cohort study by Tsukita et al. found that regular physical activity is linked to a more favorable clinical course of PD [133]. In a meta-analysis conducted by Ernst et al., the effects of various types of physical exercise in adults diagnosed with PD were compared. This study reported beneficial effects on health, with moderate-intensity exercises, including dance, being among the main activities that improved motor symptoms [134]. Studies suggest that improvements in motor symptoms and overall well-being may become apparent after 12 weeks of physical activity [135].

Similarly, regular dancing has benefits for mobility and balance in PD patients. It activates neurons that promote motor control and enhances blood flow to the frontal, temporal, parietal, and hippocampal cortices. Additionally, it regulates autonomic dysfunction, improving sympathetic cardiac regulation in PD patients. Yoga promotes balance, strength, and flexibility of the upper and lower limbs [136]. Johansson et al. demonstrated that engaging in aerobic exercise improves cognitive control by strengthening the functional connectivity between the anterior putamen and the sensorimotor cortex [137].

### 8.2. Nutrition 

Patients with PD should ensure they consume enough calcium and vitamin D through their diet or supplements, as they face a higher risk of developing sarcopenia and osteoporosis. It is also important to address constipation by maintaining adequate hydration [138]. The ketogenic diet is characterized by producing ketone bodies (acetoacetate, acetone, and β-hydroxybutyrate), inducing a state of ketosis in our body. This is achieved by increasing the energy intake from fats and reducing carbohydrate consumption. It is also achieved through fasting or a low-calorie diet. There are several studies demonstrating the role of the ketogenic diet in PD, indicating that this dietary approach has an anti-inflammatory effect, thereby reducing the inflammatory process associated with neurodegenerative diseases [139].

Nutrition, as in most conditions, plays an important role in disease progression. A Western diet characterized by high calorie intake, for example, red meats, saturated fats, processed foods, and fried foods, is associated with a poorer prognosis in neurodegenerative diseases. In contrast, the Mediterranean diet has demonstrated positive effects on longevity and overall health. This dietary pattern emphasizes a high intake of whole grains, vegetables, fruits, seeds, nuts, and legumes, and the use of olive oil. Additionally, it includes moderate consumption of saturated fats, red meats, fish, eggs, yogurt, cheese, poultry, and milk. The Mediterranean diet is rich in vitamins, minerals, antioxidants, and anti-inflammatory agents, which reduce the rate of progression of PD [140].

## 9. Palliative Care

Palliative care (PC) is a crucial component of comprehensive healthcare, dedicated to addressing the diverse forms of severe suffering that individuals experience, including physical, emotional, social, and spiritual distress. This approach is considered a universal ethical obligation [141]. Despite its importance, the World Health Organization (WHO) estimates that, globally, only 14% of patients who require palliative care actually receive it [142].

Historically, palliative care has primarily been directed towards patients with conditions such as cancer, cardiovascular disease, and major organ failure. In contrast, its application for patients with PD has been less common [143].

PD presents multifaceted challenges that extend beyond its hallmark motor symptoms. Individuals diagnosed with PD also contend with significant non-motor symptoms, including pain and dementia, which profoundly affect their quality of life and are associated with increased mortality rates [144]. Managing persistent pain in PD patients requires a comprehensive approach that considers its physical, functional, psychological, and social impacts [145].

Effective pain management strategies involve proactive measures to prevent avoidable pain. For instance, maintaining joint mobility and practicing good skin care are essential for individuals who are bedridden or chair-bound. Targeting the underlying causes of pain is prioritized in treatment efforts whenever feasible. This multifaceted approach aims not only to alleviate distress but also to enhance overall well-being throughout the course of the illness [146].

In a randomized controlled clinical trial by Kluger et al., they found that individuals who were randomly assigned to integrated palliative care exhibited enhanced quality of life, reduced symptom burden, and increased rates of advance directive completion, suggesting overall improvement. Additionally, there were indications of relief of caregiver burden and reduced hospital deaths in these patients [147].

A fundamental primary care strategy for PD entails delivering the diagnosis and prognosis with empathy, engaging in conversations about care goals and advance directives, conducting thorough evaluations of both non-motor and motor symptoms, encompassing psychosocial aspects, and making prompt referrals to hospice services when appropriate. This comprehensive approach aims to address the multifaceted needs of patients, fostering their well-being and ensuring optimal support throughout their journey with the disease [145,148] (Table 1).

## 10. Conclusions

The understanding and management of PD have undergone significant advancements in recent decades. Currently, management strategies encompass pharmacological options alongside non-pharmacological interventions. Surgical interventions are reserved for those who meet specific criteria following pharmacological therapy. Emerging therapeutic alternatives offer distinct advantages; nonetheless, there remain uncertainties regarding their comparative benefit to standard treatment and their practical application in clinical settings. Thus, further research is warranted to elucidate such factors. Therefore, it is justified to carry out more clinical research mainly on the use of antidiabetics, gene therapy, and stem cells to specify these treatments and develop our most effective strategies with a personalized approach for each patient.

## Figures and Tables

**Table 1:** Comparative analysis of therapeutic approaches in Parkinson’s disease.

Therapeutic Approaches in Parkinson’s Disease		
Conventional Treatment		
Drug/Intervention	Outcomes	References
Levodopa	Improves motor symptoms, primarily tremors and rigidity, but carries a higher risk of causing dyskinesias and motor fluctuations.	[15,53,54,55]
DA	Non-ergot dopamine agonists are not as potent as carbidopa in alleviating motor symptoms of PD, but they are less likely to induce dyskinesias or motor fluctuations. They are more effective in treating motor symptoms than MAOI-Bs, but they may cause adverse effects.	[57,58]
MAOI-Bs	Motor symptoms improve, but with less efficacy compared to levodopa; however, they present a lower risk of dyskinesia. Considered as first-line medications in younger patients with mild motor symptoms at the time of diagnosis.	[10,50,53,56]
COMT-Is	They increase levodopa levels in the CNS directly and through the reduction of its competitor 3-OMD. They exhibit notable side effects, with tolcapone in particular being associated with liver failure.	[56,59,60]
Anticholinergic agents	They are considered for initial treatment in patients under 65 years old who solely exhibit tremors; however, due to limited efficacy and adverse effects, their use is restricted.	[53,63]
Antidiabetic Drugs		
Intranasal insulin	It prevents the death of dopaminergic neurons, thereby improving motor deficits in PD. Similarly, it repairs insulin signaling pathways, thereby improving dopamine-dependent cognitive deficits.	[69,70]
GLP-1 agonists/DPP-4 inhibitors	They possess anti-inflammatory and antiapoptotic properties, which improve cognitive and motor performance. They stimulate neurogenesis.	[72,74]
Biguanide	It exerts anti-inflammatory and neuroprotective effects, which may help in slowing the progression of PD. However, it results in an increased risk of cognitive impairment and the development of PD.	[78,79,82,83,84]
Personalized Medicine		
Pluripotent stem cells	In transplanted brains with dopaminergic neurons, extensive growth and innervation of the striatum have been demonstrated.	[90]
Gene therapy	Disease modifiers: They help increase TH+ production and enhance the preservation of dopaminergic neurons.	[98,99]
	Non-modifiers of the disease: They reduce parkinsonian symptoms and the need for medication in advanced stages.	[97]
Surgical Treatments		
Unilateral thalamotomy	Controls medication-resistant tremor in some PD patients.	[108]
Unilateral pallidotomy	Effective and safe in severe dyskinesia and motor symptoms, and can be maintained for years.	[108]
Unilateral subthalamotomy	It is an experimental treatment because it presents persistent dyskinesia.	[108]
Unilateral thalamotomy with MRI-guided focal ultrasound	Given as progressive doses of ultrasound energy, which are gradually applied until ablation temperatures are reached at the target. Its objective is to reduce tremors.	[106,109]
Radioneurosurgery	Damages nervous structures through high doses of ionizing radiation, improving PD symptoms.	[106,110]
DBS	Small electrodes with a quadripolar configuration are used to release electrical impulses. The two main targets of DBS are the subthalamic nucleus and the GPi. Its objective is to treat motor symptoms.	[107,112]
Focused ultrasound stimulation (FUS)	FUS is a minimally invasive technique that precisely targets deep tissues, offering a safer alternative to invasive surgical procedures. In PD, FUS, particularly HIFU, shows promise in neuromodulation and improving motor symptoms with minimal collateral damage.	[113,114,115]
Gamma knife thalamotomy (GKT)	Gamma knife thalamotomy (GKT) is a minimally invasive neurosurgical procedure that precisely delivers gamma radiation to the ventral intermediate nucleus of the thalamus, aiming to disrupt abnormal neural circuits responsible for tremors, particularly in conditions like Parkinson’s disease.	[116,117,118]
Continuous Infusion Treatment		
LCIG	It decreases the “off” time, increases the “on” time, and may reduce dyskinesias. There are various adverse effects to consider.	[121,122,124]
Subcutaneous continuous infusion	Minimally invasive systems that increase the “on” time and decrease “off” time. Has greater solubility and concentration with lower fluctuations.	[125,126,127]
Apomorphine subcutaneous infusion	Reduces “off” time, and provides sense of improvement. Increases time spent without troublesome dyskinesia or the need for concomitant oral therapy.	[128,129,130]
Lifestyle Changes		
Exercise	Enhances functional capacity, and slows disease progression with rehabilitation and physiotherapy while improving motor symptoms in these patients.	[132,133]
Nutrition	In order to avoid osteoporosis and sarcopenia, the consumption of calcium and vitamin D is fundamental.The Mediterranean diet helps slow the progression of this disease.	[138,140]
Palliative Care		
PC interventions	Incorporating primary palliative care into the management of PD has the potential to improve the quality of life for both patients and their caregivers. This approach addresses a broad spectrum of needs, including physical, emotional, social, and spiritual aspects, and ensures timely referrals to hospice services when needed.	[142,146]

## Data Availability

Not applicable.
